# Epidemiological and Clinical Profile of Pediatric Burns in the COVID-19 Era: The Experience of a Reference Center

**DOI:** 10.3390/children9111735

**Published:** 2022-11-11

**Authors:** Gloria Pelizzo, Giulia Lanfranchi, Marcello Pantaloni, Anna Camporesi, Paola Tommasi, Eleonora Durante, Sara Costanzo, Carlotta Maria Paola Canonica, Elena Zoia, Gianvincenzo Zuccotti, Valeria Ruotolopalmi, Claudia Donzelli, Giulia Lina Tosi, Valeria Calcaterra

**Affiliations:** 1 Pediatric Surgery Department, “Vittore Buzzi” Children’s Hospital, 20154 Milan, Italy; 2 Department of Biomedical and Clinical Science, University of Milan, 20157 Milan, Italy; 3 Plastic and Reconstructive Surgery Unit, Fatebenefratelli Sacco Hospital, 20154 Milan, Italy; 4 Pediatric Intensive Care Unit, “Vittore Buzzi” Children’s Hospital, 20154 Milan, Italy; 5 Pediatric Department, “Vittore Buzzi” Children’s Hospital, 20154 Milan, Italy; 6 Head Nurse Operating Room, “Vittore Buzzi” Children’s Hospital, 20154 Milan, Italy; 7 Head Nurse Pediatric Surgery Unit, “Vittore Buzzi” Children’s Hospital, 20154 Milan, Italy; 8 Pharmacy Service Manager, “Vittore Buzzi” Children’s Hospital, 20154 Milan, Italy; 9 Pediatrics and Adolescentology Unit, Department of Internal Medicine, University of Pavia, 27100 Pavia, Italy

**Keywords:** burns, pediatrics, multidisciplinary care, prevention, children

## Abstract

Pediatric burns represent a significant public health problem. We analyzed the characteristics of pediatric burns in a reference center, in order to identify better strategies for prevention and care. Burn patients admitted to the pediatric departments of our hospital from January 2020 to June 2022 were retrospectively evaluated. Age, gender, the etiology of injuries, the total burn surface area (TBSA), the degree of burns, the length of hospital stay (LOS), concomitant SARS-CoV-2 infection, and burn surface microbial colonization were analyzed. Forty-seven patients were included in the analysis (M:F = 1:0.67). Most of the cases involved patients between 0 and 4 years of age (83%). Hot liquid burns accounted for 79% of cases, flame burns for 9%, thermal burns for 6%, scald burns for 4% and chemical burns for 2%. Mean TBSA was 14 ± 11%. A second-degree lesion was detected in 79% of patients and third-degree in 21%. Mean LOS was 17 days. No additional infection risks or major sequelae were reported in patients with SARS-CoV-2 infection. Fifteen different species of bacteria plus C. parapsilosis were isolated, while no anaerobic microorganisms were detected. In the light of our experience, we recommend a carefully planned and proactive management strategy, always multidisciplinary, to ensure the best care for the burned child.

## 1. Introduction

Approximately 11 million people worldwide suffer burn injuries each year [1]. The incidence of burns is higher in children than in adults. Almost 50% of patients are children; those under the age of 14 represent approximately one third of burn cases, 25% of them needing admission to hospital with severe lesions [2,3].

Infants’ skin is thinner and less resistant if compared with older children and adults; consequently, the same exposure can lead to more severe burns over a shorter period of time. Some factors that appear to increase the risk of burns in children have also been identified, such as a low–medium socioeconomic level, the parental education level, the child’s lower age and overcrowding of living spaces [4,5].

The majority of pediatric patients with a total burn surface area (TBSA) lower than 20% survive; however, they subsequently have to live with the physical and psychological sequelae of most burns, such as possible hypertrophic scars with the risk of reduced motility of the affected areas, sometimes associated with systemic comorbidities and mental health problems resulting from the trauma itself and the following hospitalization [6].

Moreover, we cannot overlook the fact that accesses to the emergency room for pediatric burns represents an often conspicuous financial burden for the national health system.

The COVID-19 pandemic led to school and daycare center closures for nearly 20 weeks during the lockdown in Italy. The pandemic has challenged every phase of patient care, from emergency diagnosis and management to surgical treatment and peri- and post-operative management in pediatrics. Burns incidence and care was also influenced by the Stay at Home (SHO) executive order during the COVID-19 pandemic [7]. There was a significant increase in the rate of burns in children during lockdown, associated with delayed presentation to the Emergency Department. Due to the fear of contracting COVID-19 in hospitals, patients—or their parents—tended to avoid or delay access to the emergency room, and this behavior has been observed for many conditions, including burns [8].

This paper aims to describe the epidemiological and clinical profile of pediatric burns in the COVID-19 era in a pediatric burn center in “Vittore Buzzi” Children’s Hospital (Milan, Italy), designated as a COVID-19 pediatric hub during the pandemic. Management strategies for burn centers, based on our experience, are proposed.

## 2. Materials and Methods

### 2.1. Patient Selection

Patients younger than 18 years of age with an admission diagnosis of burns at “Vittore Buzzi” Children’s Hospital, in Milan, from 1 January 2020 to 30 June 2022, were included in this study.

The “Vittore Buzzi” Children’s Hospital was identified as a COVID-19 pediatric hub at the beginning of the pandemic. It is also a third-level center for all pediatric surgical and medical emergencies, equipped with a Pediatric Intensive Care Unit (PICU) with a team of dedicated anesthetists. Children under the age of five who had severe burns (TBSA > 15%) and/or who needed PICU were admitted according to Regional Burn Center guidelines.

A Burn Care Team was created, comprising two senior pediatric surgeons and two nurses trained in pediatric burn management. The entire team included professionals from the Pediatric Surgery Department, the Pediatric Department, the Anesthesiology Unit and the Emergency Department. Pediatric plastic surgeons were also involved, to ensure adequate treatment and training, in accordance with national guidelines for burns.

### 2.2. Data Collection

The following data were collected from the medical records: age, gender, etiology of injuries, admission date, total burn surface area (TBSA), degree of burns, length of hospital stay (LOS), number of dressings required, SARS-CoV-2 infection and burn microbial colonization.

The Lund–Browder classification was used to calculate the affected body surface area of the patients. Patients were divided into four groups according to TBSA, small area (<10% TBSA), medium area (10–20% TBSA), large area (20–30% TBSA) and extra-large area (>30% TBSA), and also classified into 3 groups according to burn degree (first-, second- and third-degree).

The treatment protocol of burns was established in accordance with the main international standards of treatment, including fluid resuscitation, antibiotic therapy, wound care, nutritional support and surgical operations.

The microbial colonization of all wounds was studied when macroscopic local signs of burn infection were present. Swabs were obtained from the lesions prior to any cleansing.

### 2.3. Statistical Analysis

Categorical variables were presented as numbers and percentages. All continuous values with normal distribution were reported as mean ± standard deviation (SD) and data were tested for normality using the Shapiro–Wilk test.

Continuous variables with asymmetric distribution were expressed as the median and the respective range interval.

Linear regression and the Pearson correlation coefficient r were used to analyze correlations between the number of dressings and TBSA and between the number of dressings and degree of burn.

Correlations were classified as strong (r > 0.70), good (0.50 < r < 0.70) or weak (0.30 < r < 0.50). A *p* value < 0.05 was considered statistically significant.

Data were analyzed with Excel 2016 software (Microsoft, Redmond, WA, USA).

## 3. Results

### 3.1. Patient Characteristics

From 1 January 2020 to 30 June 2022, a total of 164 pediatric patients (97 males, 67 females) presented to our center following accidental burns.

One hundred and seventeen patients (71%; 69 males, 48 females) had reported superficial lesions and could be treated in the Emergency Room and were observed for less than eight hours before being discharged home.

**Figure 1:**
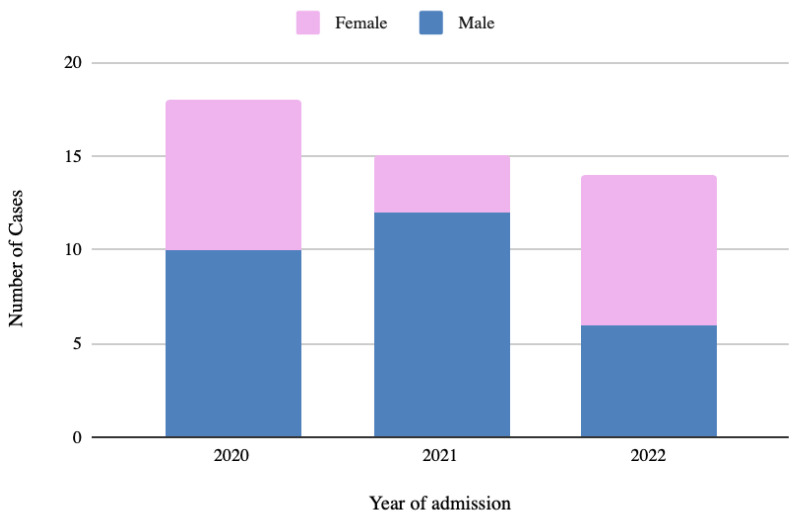
Number of pediatric burn patients needing hospital admission, divided by year.

Of the 164 subjects, 47 (29%) met the inclusion criteria for admission to the Burn Service and 18 (11%) were admitted to the Pediatric Intensive Care Unit (PICU) due to the extent of their lesions (more than 20% TBSA). The number of admitted patients per year is summarized in Figure 1.

General demographics are shown in Table 1.

### 3.2. Distribution by Age and Gender

**Figure 2:**
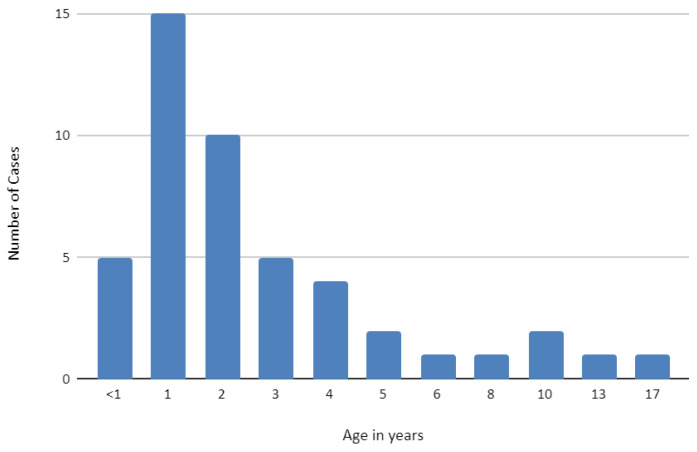
Age distribution of all pediatric burn patients.

At the time of burn, the median age was 2.1 years, ranging from 4 months to 17 years. The majority of children (*n* = 34, 83%) were between 0 and 4 years old, followed by 5 to 8 years (9%) and 9 to 12 years (4%). Only 4% of the patients were older than 13 years (Figure 2). In patients aged 0 to 8 years, contact burns were the most common etiology. Males sustained more often contact burns (63.5% vs. 37.5%), compared to females.

### 3.3. Distribution of Etiology

**Figure 3:**
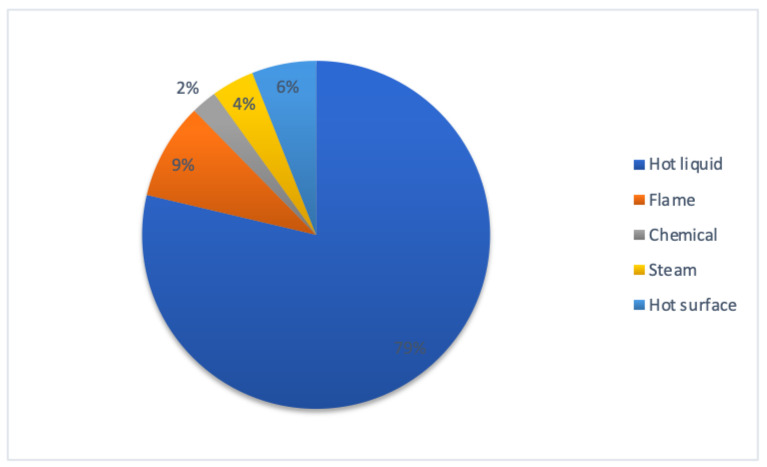
Percentage of different etiologies of pediatric burns.

The leading cause was hot liquid burns (37 patients, 79%), followed by flame burns (4, 9%), hot surface burns (3, 6%), steam burns (2, 4%) and chemical burns (1, 2.4%) (Figure 3). There were significant differences in age among different etiology groups, with the mean age of flame burn patients being 8.5 years; that for contact burns was 3.3 years, that for chemical burns was 3 years and that for scalds was 3.2 years.

### 3.4. Distribution of Total Burn Size Area (TBSA)

The average TBSA was 14% ± 11% (range 2–70%). Fifty-one percent of the lesions were medium and small area burns. There were a few large (25% TBSA) and extra-large (35% TBSA) area burns, the majority caused by flames. No significant relationship was observed between hot water burns and gender (*p* > 0.05).

### 3.5. Distribution of the Degree of Burns

**Figure 4:**
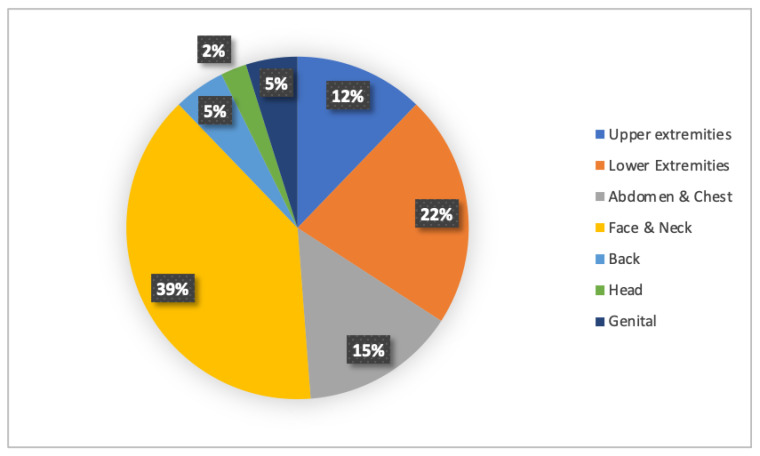
Anatomical locations of pediatric burns among patients.

Distribution of the degree of burns was 37 (78%) second-degree and 10 (2%) third-degree burns. Third-degree burns were observed more frequently in the 4 months–2 years age range, with different etiologies (chemical burns, flame burns and contact burns), the most frequent being hot liquid burns and chemical burns (35%). Anatomically, the majority of the burns were confined to the head, face and neck (17; 36%), followed by burns of the lower extremities (10; 21%), as shown in Figure 4. There were significant differences among ages for different anatomical locations, with the medium age of face and neck burns being 2.4 years; that for lower extremity burns was 5.1 years, and that abdomen and chest burns was 5.6 years. The features of patients are reported in Table 2.

### 3.6. Distribution of Length of Stay (LOS)

The minimum length of stay (LOS) was one day, while the maximum was 69 days. The average LOS was 17 ± 15 days, with a prevalence of hot liquid burns in the long LOS group.

There were significant differences among TBSA groups for LOS, with a mean LOS of 44 days for the extra-large area group, 18 days for the large area group, 16 days for the medium area group and 9 days for the small area group.

### 3.7. Number of Dressings

**Figure 5:**
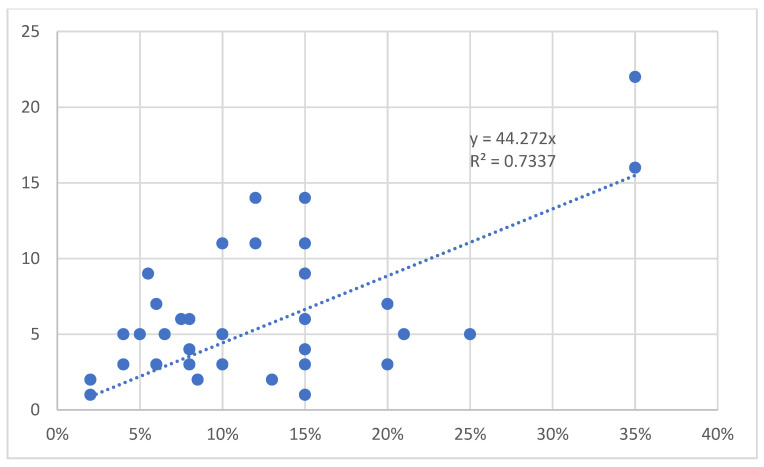
Correlation between number of dressings and extent of the lesion.

Wound dressing was the primary treatment for all burn wounds. Nine patients (19%) also underwent surgical procedures. Debridement and skin grafting were highly correlated with cause, age, full-thickness burns and TBSA among children with burns. The percentage and frequency of dressings did not significantly differ between boys and girls (*p* > 0.05) The average number of dressings was 6 ± 4. A correlation was found between the number of dressings and TBSA (r = 0.73, *p* < 0.05) (Figure 5). A correlation was also found between TBSA, depth of burn and dressings (r = 0.72, *p* < 0.05).

### 3.8. SARS-CoV-2 Infection

At admission, four patients tested positive on the SARS-CoV-2 nasopharyngeal swab; three parents also tested positive on admission until discharge. During hospitalization, two patients developed positivity on the SARS-CoV-2 nasopharyngeal swab.

No transmission was recorded between SARS-CoV-2-positive and -negative patients in the ward and PICU. No transmission was recorded between patients and healthcare professionals. Treatment of SARS-CoV-2-infected pediatric burn patients was not burdened with additional risks of infection or major sequelae.

During the lockdown and SHO period, we noted delayed presentation (> 24 h after burn) to the Emergency Department in 19% of the patients.

### 3.9. Burn Microbial Colonization

The study consisted of 34 samplings in the form of surface swabs obtained from the burn wounds on occasion of dressings or surgical procedures, of which eight yielded no growth. The total number of microbial isolates was 45 (Table 3). Fourteen different species of bacteria and only one species of fungi (Candida parapsilosis) were recovered in cultures. No anaerobic organisms were detected.

Culture from burn swabs revealed a prevalence of Gram-positive organisms, as they accounted for 67% of all isolates.

## 4. Discussion

Pediatric burns are potentially severe injuries, often resulting in significant morbidity and impaired emotional well-being and quality of life. In addition to immediate and often stressful care, burns often require prolonged treatment, with repeated dressing changes, prolonged hospital stays and multiple reconstructive surgical procedures. In addition, daily and impactful practices such as wearing customized compression garments and regular physiotherapy are often required [9].

Our hospital is a third-level pediatric burn center accepting patients from a large area of the country. The number of patients referred from other cities increased after the Burn Center became active in January 2020.

In our study, young age was the most relevant risk factor for burns, with 75% of cases having occurred in patients younger than 4 years of age. The prevalence of burns in this study was inversely related to age. These data were similar to those of other studies [10,11].

In our study, hot fluids were the most common cause of burns in small children, while school-aged children were more commonly exposed to flame burns. In the literature, the most commonly reported injury mechanism is hot liquid scald, which usually occurs in the home environment in children younger than 5 years old [12].

In our population, the face and neck (36%) was the most affected area, followed by the lower extremities (22%); this finding is different from those of some other published series, in which burns occur most frequently in the extremities [13,14]. The upper limbs were the most involved in 90.42% of the cases, followed by the lower limbs (80.85%), as the study showed [15]. This could be explained considering the ages of the children involved. When older children accidentally touch a container of hot liquid, pulling it from a stove or work surface, the consequence can be immersion or spillage of the hot substance, usually on the extremities of the children. On the opposite side, children younger than 4 years are usually accidentally exposed to hot liquid and this results in burns of the face–neck area.

A significant relationship was found between TBSA and the degree of the burn, number of surgical interventions and development of infections, as well as the number of days of hospitalization.

Surgery is a reliable means of removing necrotic tissue from the wound, thus improving the patient’s prognosis. The wound is completely debrided, thus reducing the inflammation, shortening the wound healing time and achieving more beneficial outcomes. Patients with full-thickness burns underwent surgery more frequently than those without full-thickness burns.

The management of deep partial and full-thickness skin defects remains a significant challenge in pediatrics. Children, in fact, present with higher rates of burn injuries, and surgical treatment in the smallest patients is demanding due to the marked donor site shortage [16].

The donor site limitations cause delays in definitive coverage and consequently may lead to sepsis, multiple organ dysfunction and higher morbidity and mortality. Moreover, children usually experience growth with unyielding scars that could lead to the risk of functionally debilitating and devastating hypertrophic scarring [17,18,19,20]. According to our experience, a good approach to severe burns is the early application of biosynthetic skin dressings or bioengineered skin substitutes. These matrices act as an effective wound temporizer while the donor sites heal and become available for further re-cropping.

We evaluated the microbial colonization pattern of the burn wound. Staphylococcus aureus was the most frequent microbial isolate (48%). This is in line with previous experiences, which also indicate a much higher frequency of isolation of this organism [21,22,23]. Regarding antimicrobial sensitivity, the antibiograms identified only one case of methicillin-resistant Staphylococcus aureus (MRSA), also in line with the antibiograms of other burn centers [24]. However, the evidence that MRSA could become a significant problem in the management of burn injuries should not be underestimated [25].

Escherichia coli was the second most frequently encountered microorganism in the present study (13%). This figure is higher than that reported by other burn centers, in which the isolation frequency of this microorganism did not exceed 5% [26,27].

In our series, Klebsiella pneumoniae followed Escherichia coli in the list of burn wound isolates, as it accounted for 7%.

We also found a low frequency of colonization by Pseudomonas aeruginosa (4%), differently from what was reported by other burn centers, where this organism was believed to be responsible for the most severe infections of burn wounds [28,29].

In the case of symptoms and signs of sepsis due to colonization of the burned area, in the absence of an isolated germ, treatment success largely depends on the timely administration of empiric intravenous antimicrobial therapy. It is therefore essential that each burn institution determine its own specific burn wound microbial colonization pattern and antimicrobial resistance profiles. This would allow for the early management of septic episodes with appropriate empiric systemic antibiotics before microbiological culture results become available, thereby improving infection-related morbidity and mortality.

Due to the progress of burn management in many fields, such as fluid resuscitation, infection control, wound management, metabolism and nutritional treatments, mortality in child burns has decreased significantly. In our series, we did not record any deaths, probably due to the rapidity of access to intensive care and the possibility of immediately benefiting from a multidisciplinary approach within a third-level pediatric hospital. Mortality rates of 0.65% to 15.4% have been reported, with higher rates in the case of flame burns [30]. The most common cause of mortality is infection, followed by systemic inflammatory response syndrome. Toxic shock syndrome (TSS) represents a severe, toxin-mediated illness that, in pediatrics, can appear even at a low degree of TBSA scald (<7%) and can mimic several other diseases. Early symptoms include fever and hypotension rash and may lead to multiorgan system involvement. The early detection of the disease may avoid a poor prognosis.

Severe burns induce, in children, an increased metabolic rate and a pathophysiological stress response that can persist for a long time after injury, and delayed nutrition and surgical excision in children result in a significant increase in patients’ energy expenditure after the initiation of treatment. Trauma and sepsis induce a condition of hypermetabolism due to metabolic, hormonal and inflammatory dysregulation.

Burned children requiring Intensive Care Unit admission should receive early enteral nutritional support and early surgical treatment, including wound excision, in order to limit the hypermetabolic response to burn [31].

A dedicated analysis of the metabolic changes in children after burns should be performed to define the ideal intensive care treatment and surgical timing.

In pediatrics, there is a diversity of burn accidents and resulting injuries. We confirm that burn etiology is age-dependent and children aged less than 5 years represent the age group with the highest risk [16]. The target groups and subjects for prevention should be better specified to optimize wound healing. A dedicated pediatric assessment and management for each burned child group could decrease morbidity and long-term complications.

It is already documented how the incidence of burns is significantly related to the level of social and economic development [9]. Pediatric burns often occur within the context of a lack of attention towards minors, due to sub-optimal socio-economic conditions. Risk factors associated with socio-economic status are difficult to eliminate in the short term and require large-scale interventions, also promoted by government agencies.

In accordance with other studies [7,8], we reported a significant increase in the rate of burn injuries during the pandemic period. The severity of burns was higher during the lockdown, as evidenced by an increase in burn alerts, TBSA, the proportion of children with >5% TBSA and Intensive Care Unit admissions.

Additionally, we noted a delay in emergency room presentation in 19% of patients. Late access to medical care causes delays in fluid resuscitation, burn wound treatment, pain control and wound infection containment, thus affecting prognosis. It has been noted that morbidity is greatest for children admitted to hospital more than four hours after the accident. Although late hospital presentation following a burn injury increases the probability of a longer hospital stay and complications [32], in our study, patients who presented later stayed for a shorter time than those who arrived earlier.

The explanation for this phenomenon can be ascribed to the minor extent of the injuries, which led the parents to bring their children to medical attention later.

The main limitation of the study is its retrospective nature. We acknowledge that our results are limited by the single-center design.

Our future perspectives cover various aspects: the continuous study of our data in order to improve the knowledge of the characteristics of our patients and the statistical significance of our acquisitions; the definition of standardized recommendations and protocols that can be shared with other burn centers; the thorough study of techniques and materials in order to guarantee, within defined paths, the adaptation and tailoring of the treatment to the individual patient.

## 5. Conclusions

Burn injuries in children may lead to serious physical and psychological sequelae in acute and chronic settings.

Our experience confirms that younger children are at risk of more severe and extensive skin lesions. For these patients, a dedicated multidisciplinary pediatric team, a high-level pediatric facility endowed with a pediatric ICU and dedicated pathways may represent a life-saving therapy.

Epidemiological studies should be strengthened to more adequately recognize and thus prevent risk factors associated with pediatric burns. Considering our experience, we recommend stronger education and prevention strategies in the population, and carefully planned treatment paths, based on a multidisciplinary team in a high-level pediatric facility endowed with a pediatric ICU. A national pediatric burn database would also be necessary to classify all types of pediatric lesions and to provide more data for tailored surgical treatments. A stronger education and prevention strategy among the population is recommended.

## Figures and Tables

**Table 1:** Features of pediatric burn patients who presented to our Emergency Unit from 1 January 2020 to 30 June 2022.

	Year 2020 (12 Months)	Year 2021 (12 Months)	Year 2022 (6 Months)
Demographics			
Total number of patients, n	18	15	14
Sex (M/F)	10/8	12/3	6/8
Age (months)	37 ± 31	46 ± 60	37 ± 33
Degree of burn n			
First	0	0	0
Second	16	11	10
Third	2	4	4
Total burn surface			
<10%	6	5	7
10–20%	10	6	5
20–30%	1	3	0
>30%	1	1	2

**Table 2:** Features of the burns.

	Number of Patients (%)
Burn degree, n (%)	
First	0 (0%)
Second	37 (78%)
Second and third	10 (22%)
Source, n (%)	
Water	35 (75%)
Oil	4 (8%)
Acid	3 (6%)
Flame	2 (4%)
Oven	1 (2%)
Coal	1 (2%)
Thermal	1 (2%)
Burn Locations, n (%)	
Upper extremities	5
Lower extremities	10
Abdomen and chest	8
Face and neck	17
Back	2
Head	1
Genital	2

**Table 3:** Pathogens identified in blood cultures from 22 burn patients.

Pathogen	Number of Patients (%)
Staphylococcus aureus	22 (48%)
Escherichia coli	6 (13%)
Klebsiella pneumoniae	2
Klebsiella ESBL+	1
Pseudomonas aeruginosa	2
Enterococcus faecalis	2
Streptococcus agalactiae	1
Streptococcus pyogenes	1
Staphylococcus epidermidis	1
Staphylococcus haemolyticus	1
MRSA	1
Candida parapsilosis	1
Enterococcus cloacae	2
Enterococcus cloacae complex	1
Proteus vulgaris	1
Isolates	45
Sampling occasions	34
Patients	22

## Data Availability

The data presented in this study are available on request from the corresponding author.
